# Randomized phase III trial of amrubicin/cisplatin versus etoposide/cisplatin as first-line treatment for extensive small-cell lung cancer

**DOI:** 10.1186/s12885-016-2301-6

**Published:** 2016-04-09

**Authors:** Yan Sun, Ying Cheng, Xuezhi Hao, Jie Wang, Chengping Hu, Baohui Han, Xiaoqing Liu, Li Zhang, Huiping Wan, Zhongjun Xia, Yunpeng Liu, Wei Li, Mei Hou, Helong Zhang, Qingyu Xiu, Yunzhong Zhu, Jifeng Feng, Shukui Qin, Xiaoyan Luo

**Affiliations:** Department of Internal Medicine, Cancer Institute & Hospital, Chinese Academy of Medical Sciences & Peking Union Medical College, Beijing, China; Department of Internal Medicine, Jilin Cancer Hospital, Jilin, China; Department of Medical Oncology, Beijing Cancer Hospital, Beijing, China; Department of Respiratory Medicine, Xiangya Hospital of Central-South University, Hunan, China; Department of Pulmonary Medicine, Shanghai Chest Hospital, Shanghai, China; Department of Lung Cancer Medicine, 307th Hospital of the Chinese People’s Liberation Army, Beijing, China; Department of Respiratory Medicine, Peking Union Medical College Hospital, Chinese Academy of Medical Sciences, Beijing, China; Department of Medical Oncology, Jiangxi Provincial People’s Hospital, Jiangxi, China; Department of Medical Oncology, Affiliated Cancer Hospital of Sun Yat-sen University, Guangdong, China; Department of Medical Oncology, The First Hospital of China Medical University, Liaoning, China; Department of Medical Oncology, The First Hospital of Jilin University, Jilin, China; Department of Medical Oncology, West China Hospital, Sichuan University, Sichuan, China; Department of Medical Oncology, Tangdu Hospital of the Fourth Military Medical University, Shanxi, China; Department of Respiratory Medicine, Shanghai Changzheng Hospital, Shanghai, China; Department of Medical Oncology, Beijing Chest Hospital, Beijing, China; Department of Medical Oncology, Jiangsu Cancer Hospital, Jiangsu, China; Department of Medical Oncology, 81st Hospital of the Chinese People’s Liberation Army, Jiangsu, China; Medical Division, Sumitomo Pharmaceuticals (Suzhou) Co., Ltd., Beijing, China

**Keywords:** Amrubicin, Cisplatin, Etoposide, ED-SCLC, Randomized clinical trial, Chinese

## Abstract

**Background:**

Extensive-disease small-cell lung cancer (ED-SCLC) is characterized by rapid progression and relapse, despite high initial response rates to chemotherapy. The primary objective of this trial was to demonstrate the non-inferiority of amrubicin and cisplatin (AP) combination therapy compared with the standard first-line regimen of etoposide and cisplatin (EP) for previously untreated ED-SCLC in a Chinese population. When non-inferiority was verified, the objective was switched from non-inferiority to superiority.

**Methods:**

From June 2008 to July 2010, 300 patients were enrolled and randomly assigned at a 1:1 ratio to AP and EP groups. AP-treated patients received cisplatin (60 mg/m^2^, day 1) and amrubicin (40 mg/m^2^, days 1–3) once every 21 days. EP-treated patients received cisplatin (80 mg/m^2^, day 1) and etoposide (100 mg/m^2^, days 1–3) once every 21 days. Treatment was continued for four to six cycles, except in cases of progressive disease or toxicity, and patient refusal.

**Results:**

Median overall survival (OS) for AP *vs.* EP treatment was 11.8 *vs.* 10.3 months (*p* = 0.08), respectively, demonstrating non-inferiority of AP to EP (AP group: 95 % confidence interval for hazard ratio 0.63–1.03 months). Median progression-free survival and overall response rates for AP *vs*. EP groups were 6.8 *vs.* 5.7 months (*p* = 0.35) and 69.8 % *vs*. 57.3 %, respectively. Drug-related adverse events in both groups were similar, with neutropenia being the most frequent (AP 54.4 %; EP 44.0 %). Leukopenia, pyrexia, and fatigue were more prevalent in the AP group, but all were clinically reversible and manageable.

**Conclusions:**

AP therapy demonstrated non-inferiority to EP therapy, prolonging OS for 1.5 months, but this difference was not statistically significant; thus we propose AP as a promising treatment option for ED-SCLC in China.

**Trial registration:**

This trial was registered on 10 April 2008 (ClinicalTrials.gov NCT00660504).

**Electronic supplementary material:**

The online version of this article (doi:10.1186/s12885-016-2301-6) contains supplementary material, which is available to authorized users.

## Background

Lung cancer is the most common cancer in China, with new cases estimated at a rate of 46.08 per 100,000 in 2010 [1]. Small-cell lung cancer (SCLC) is the most aggressive subtype, accounting for approximately 15–20 % of lung cancers and is classified as limited or extensive disease [2, 3]. Extensive-disease (ED)-SCLC accounts for 60–70 % of all SCLC cases and is characterized by rapid progression [4]. SCLC is chemosensitive and combination chemotherapy is effective for cases of untreated ED-SCLC, but only 15–20 % of patients achieve a complete response; most eventually relapse, and the median survival time (MST) from diagnosis is only 9–10 months. Combination chemotherapy using a platinum-based drug plus etoposide is the most commonly used regimen for first-line treatment for metastatic SCLC, and etoposide plus cisplatin (EP) therapy has been the global standard since the mid-1980s [5–7]. Over the last two decades, many regimens of targeted therapies and newer chemotherapeutic agents have been trialed [8–16], but the outcome for SCLC patients has not been significantly improved.

Amrubicin is a synthetic anthracycline and a potent topoisomerase II inhibitor. Its acute toxicity is qualitatively similar to that of doxorubicin, but amrubicin shows almost no heart damage at cumulative doses [17, 18] and does not exhibit the chronic cardiotoxic effects (e.g., congestive heart failure) in rabbits and dogs that are observed with doxorubicin [19–21].

In 2002, amrubicin was approved for NSCLC and SCLC treatment in Japan, and shows promising efficacy as a single agent therapy. In a phase II study, 33 previously untreated ED-SCLC patients received amrubicin monotherapy with a dose schedule of 45 mg/m^2^ on days 1–3 every 3 weeks. The overall response rate (ORR) was 75.8 %, the MST was 11.7 months, and the 1-year survival rate was 48.5 % [22]. Amrubicin also showed good efficacy when administered in combination with platinum. In a phase I/II study in 41 previously untreated patients, the ORR was 87.8 %, the MST was 13.6 months, and the 2-year survival rate was 17.6 % [23]. Further phase II studies have been conducted in Western populations with initial ED-SCLC. In one such study, 30 patients received amrubicin with cisplatin. The ORR was 76.7 % and the MST was 11.1 months [24]. A phase II study of amrubicin as second-line therapy in 75 patients with platinum-refractory SCLC enrolled from the US and EU revealed an ORR of 21.3 %, and median progression-free survival (PFS) and overall survival (OS) times of 3.2 months and 6.0 months, respectively, in parallel with an acceptable safety profile [25]. Similarly, in another phase II study conducted in Western patients (*n* = 76) in which amrubicin was compared with topotecan, amrubicin achieved a significantly higher ORR of 44 % and had a similar safety profile to topotecan [26]. A recent second-line phase III trial in 637 patients recruited from the US, Europe, and Australia showed that amrubicin did not improve survival, but that it had demonstrable activity and a good safety profile compared with that of topotecan [27].

Here, we report the results of multicenter, open-label, randomized phase III trial comparing amrubicin and cisplatin (AP) therapy with EP therapy in previously untreated Chinese ED-SCLC patients. The primary objective of this trial was to demonstrate non-inferiority in OS, and when non-inferiority was verified, the objective was switched from non-inferiority to superiority.

## Methods

### Study design and patients

This multicenter, randomized, phase III, open-label study involved 17 Chinese hospitals. Patients with histologically or cytologically documented SCLC were eligible for inclusion. Each patient was required to meet the following criteria: extensive-stage disease; no prior therapy for the primary lesion; a measurable lesion; Eastern Cooperative Oncology Group performance status (ECOG PS) of 0 or 1; age ≥18 years; adequate hematological function (white blood cells ≥4,000–10,000/μL, neutrophils ≥2,000/μL, blood platelets ≥100,000/μL, hemoglobin ≥9.5 g/dL); adequate hepatic function (aspartate aminotransferase and alanine aminotransferase ≤2.5-fold upper limit of normal, serum bilirubin <1.5-fold upper limit of normal, adequate renal function (serum creatinine ≤ upper limit of normal); minimum life expectancy ≥3 months; no electrocardiogram abnormality requiring treatment; left ventricular ejection fraction (LVEF) ≥55 %; and provision of written informed consent. Patients with known brain metastasis were eligible if they were asymptomatic and had stable disease without any therapy.

Patients were excluded if they had received any previous therapy for the primary lesion, pleural effusion requiring drainage, superior vena cava syndrome, gastric or duodenal ulcers, severe heart disease, severe renal disease, active concomitant malignancy, symptomatic pneumonitis, or pulmonary fibrosis. Pregnant or nursing women were also excluded.

The protocol was conducted in accordance with the Declaration of Helsinki, and was approved by the institutional ethics committee at each center (a list of ethics committees is provided in the Supporting Information [Additional file 1]), and all patients initially provided written informed consent. The study was registered at ClinicalTrials.gov (NCT00660504).

### Randomization

Patients who met the entry criteria were registered and randomly assigned to a treatment centrally via an interactive web response system. Patients were assigned at a 1:1 ratio to AP or EP treatment groups using a computer-generated randomization list. Central random assignment by dynamic allocation to the AP or EP group was stratified according to institution, sex, and ECOG PS (0 or 1), and was balanced for stratification factors using the Pocock and Simon dynamic balancing procedure [28].

### Treatments

Treatment commenced within 14 days of randomization. Based on the result of a Japanese Phase I/II study [23], patients in the AP group received cisplatin (60 mg/m^2^, day 1) and amrubicin (40 mg/m^2^, days 1–3) once every 21 days. Those in the EP group received the Chinese standard regimen of cisplatin (80 mg/m^2^, day 1) and etoposide (100 mg/m^2^, days 1–3) once every 21 days. Patients were treated for 4–6 cycles until the occurrence of progressive disease or toxicity, or patient refusal. Dose modifications were allowed in cases of toxicity. The amrubicin dose was reduced in increments of 5 mg/m^2^/day for grade 3 or 4 neutropenic fever or sepsis, grade 4 neutropenia lasting ≥4 consecutive days, grade 4 thrombocytopenia, or any grade 3 or 4 nonhematologic toxicity except nausea or vomiting; the etoposide dose was reduced in increments of 20 mg/m^2^/day for patients exhibiting the same symptoms. The cisplatin dose was reduced in increments of 20 mg/m^2^/day for serum creatinine escalation, grade 3 nonhematologic toxicity except nausea or vomiting, and neuropathic disorders. Once a dose reduction had been implemented, the dose could not be re-escalated. Following treatment, prophylactic cranial irradiation (PCI) was offered to patients who had achieved complete response or good partial response.

### Endpoints

The primary endpoint was OS, and the secondary endpoints were PFS and investigator-determined ORR. Tumor response was evaluated using Response Evaluation Criteria In Solid Tumors version 1.0. The antitumor effect was evaluated by computed tomography every two cycles after the first injection. Stable disease was defined as a case that met the defined criteria for stable disease at least twice after study entry at a minimum interval of 6 weeks. Adverse events (AEs) were graded according to the Common Terminology Criteria for AEs version 3.0; no cutoff period was defined for treatment-emergent AEs.

### Statistical analysis

An intention-to-treat (ITT) analysis, which excluded one patient who withdrew consent before the first administration, was used for efficacy and safety analyses. Patient sample size was determined by taking into account the enrollment period of 1.5 years and the commencement of follow-up at 1.5 years after the last patient enrollment, with a two-sided significance value of 5 % (95 % confidence interval (CI) for evaluation). MSTs of 13.6 months [23] and 9.4 months [22] were assumed for AP and EP therapy of ED-SCLC, respectively. Non-inferiority was defined by the upper limit of the 95 % CI for the hazard ratio (HR) being set at <1.25. Non-inferiority was established with >99 % power for 300 patients (150 per group) in total. Superiority could be determined if the upper limit of the 95 % CI for HR was <1, and could be confirmed following validation of non-inferiority. Under these conditions, superiority could also be calculated with a power of more than 80 %. The point estimate and 95 % CI of the HR for the AP group relative to the EP group were calculated for OS and PFS with the Cox proportional hazards model using the following factors (excluding the clinical trial institution) defined during dynamic randomization: treatment; PS at baseline; and sex.

## Results

### Patients

**Fig. 1:**
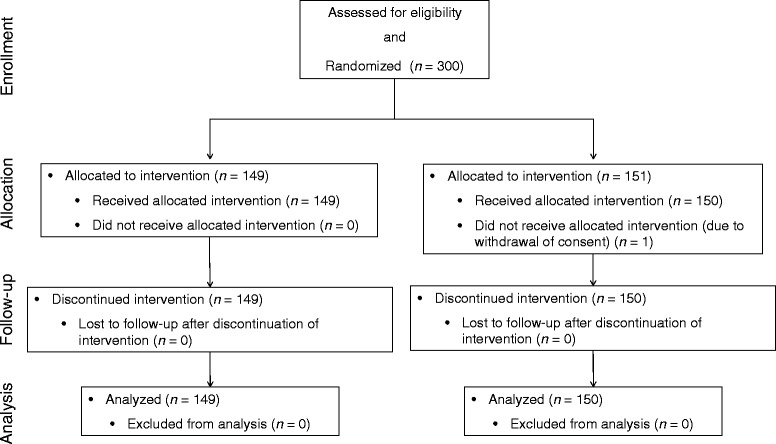
Patient flowchart. AP, amrubicin/cisplatin; EP, etoposide/cisplatin

From June 2008 to July 2010, 300 patients were enrolled and randomly assigned at a 1:1 ratio to the AP and EP groups (Fig. 1). One patient withdrew informed consent before administration and was excluded from the ITT analysis. Therefore, 299 patients (AP group, *n* = 149; EP group, *n* = 150) were included in the ITT analysis of efficacy and safety. The baseline demographic and disease characteristics of the patients are listed in Table 1. There was no difference in patient baseline demographics between the groups.

**Table 1:** Patient characteristics

	AP group	EP group
Number of patients	149	150
Sex		
Male	114 (76.5 %)	113 (75.3 %)
Female	35 (23.5 %)	37 (24.7 %)
Median age, years (SD)^a^	58.0 (13.0)	59.0 (13.0)
ECOG PS		
0	42 (28.2 %)	32 (21.3 %)
1	107 (71.8 %)	118 (78.7 %)
Stage		
IIIB	3 (2.0 %)	9 (6.0 %)
IV	146 (98.0 %)	141 (94.0 %)
Metastasis^b^	148 (99.3 %)	144(96.0 %)
Lung	18 (12.1 %)	22 (14.7 %)
Bone	60 (40.3 %)	65 (43.3 %)
Brain	30 (20.1 %)	17 (11.3 %)
Liver	37 (24.8 %)	41 (27.3 %)
Other	82 (55.0 %)	78 (52.0 %)

Data are number (%) except ^a^median (SD)

^b^Several patients had metastases to multiple sites

*AP* amrubicin/cisplatin, *EP* etoposide/cisplatin, *SD* standard deviation, *ECOG PS* Eastern Cooperative Oncology Group

### Treatment delivery

The median numbers of treatment cycles were 4.6 in the AP group and 4.5 in the EP group; 118 patients in the AP group and 110 in the EP group completed four to six cycles. During the study period, 90 patients in the AP group and 73 patients in the EP group received a dose reduction or had their treatment schedule prolonged. Although eight patients in the AP group and one in the EP group needed two dose level reductions (amrubicin; 30 mg/m^2^/day, etoposide; 60 mg/m^2^/day), almost all patients received >80 % of the planned dosage. Thirty-one patients in the AP group and 41 in the EP group were withdrawn from treatment, mainly because of patient request (AP group, nine; EP group, 11) and disease progression (AP group, six; EP group, 13).

### Efficacy

#### Survival

**Fig. 2:**
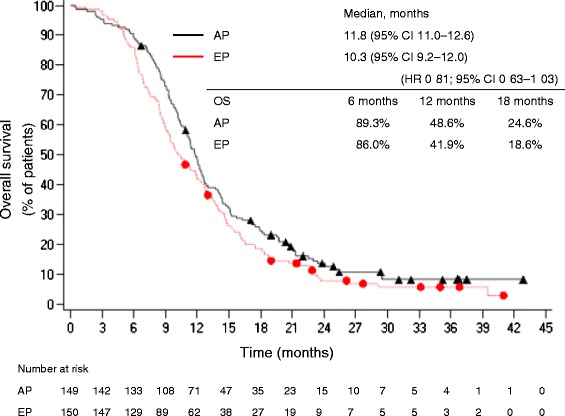
Cumulative survival rate of patients. AP group (*n* = 149; black triangles), EP group (*n* = 150; red circles) (ITT population). AP, amrubicin/cisplatin; CI, confidence interval; EP, etoposide/cisplatin; HR, hazard ratio; OS, overall survival

The primary endpoint of OS is shown in Fig. 2. The final survival follow-up point was defined as 1.5 years after enrollment of the last patient. The median OS (95 % two-sided CI) was 11.8 months (range, 11.0–12.6 months) in the AP group and 10.3 months (range, 9.2–12.0 months) in the EP group. Therefore, the AP group demonstrated non-inferiority to the EP group, in as much as the HR was 0.81 and the 95 % CI was 0.63–1.03, which met the criteria for non-inferiority. Additionally, regarding the analysis for superiority, the AP group showed an improved median OS that was 1.5 months longer than that of the EP group, but this difference was not statistically significant (*p* = 0.08). The 1-year survival rates in the AP *vs*. EP groups were 48.6 % (95 % CI 40.3–56.4) *vs.* 41.9 % (95 % CI 34.0–49.7), respectively.

#### PFS

**Fig. 3:**
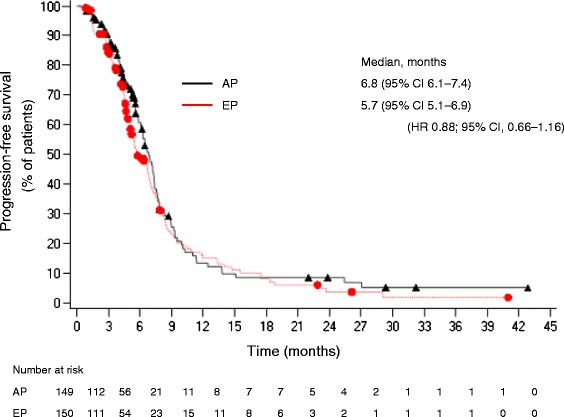
Progression-free survival of patients. AP group (*n* = 149; black triangles), EP group (*n* = 150; red circles) (ITT population). AP, amrubicin/cisplatin; CI, confidence interval; EP, etoposide/cisplatin; HR, hazard ratio; ITT, intent-to-treat

PFS was a secondary endpoint in this study (Fig. 3). The median PFS was 6.8 months (range, 6.1–7.4 months) in the AP group and 5.7 months (range, 5.1–6.9 months) in the EP group (HR for AP 0.88; 95 % CI 0.66–1.16) but these differences were not statistically significant (*p* = 0.35).

#### ORR

ORRs were 69.8 % (104/149) in the AP group and 57.3 % (86/150) in the EP group. Five complete and 99 partial responses were achieved in the AP group, while the EP group demonstrated three complete and 83 partial responses. The ORR in the AP group was significantly improved compared with that in the EP group (95 % CI 1.7–23.3 %).

### Safety

The AEs observed during this study are listed in Table 2. The most common AE of grade 3 or worse in both groups was hematologic toxicity: in the AP and EP groups, neutropenia occurred in 54.4 % (81/149) and 44.0 % (66/150) of patients, respectively, leukopenia in 34.9 % (52/149) and 19.3 % (29/150), respectively, and thrombocytopenia in 16.1 % (24/149) and 7.3 % (11/150), respectively. The AEs with absolute >10 % differences between the two groups (AP *vs.* EP) were pyrexia (18.8 *vs.* 8.0 %), fatigue (18.1 *vs*. 7.3 %), and diarrhea (16.8 *vs.* 8.7 %). These incidences were higher in the AP group, but most cases recovered and the AEs were manageable for both groups. Six patients in the AP group had febrile neutropenia, but no cases were observed in the EP group.

**Table 2:** Hematological and non-hematological adverse events

	AP group (*n* = 149)				EP group (*n* = 150)			
	Total		≥Grade 3		Total		≥Grade 3	
Events (CTCAE v3.0)	*n*	%	*n*	%	*n*	%	*n*	%
Patients with one or more adverse events	149	100	-	-	148	98.7	-	-
Anemia	48	32.2	10	6.7	48	32.0	10	6.7
Hemoglobin decreased	49	32.9	16	10.7	50	33.3	8	5.3
Leukopenia	97	65.1	52	34.9	85	56.7	29	19.3
Neutropenia	99	66.4	81	54.4	85	56.7	66	44.0
Thrombocytopenia	54	36.2		16.1	40	26.7	11	7.3
Constipation	32	21.5		0	25	16.7		0
Diarrhea	25	16.8	3	2.0	13	8.7	1	0.7
Gastrointestinal disorder	22	14.8	3	2.0	26	17.3	1	0 · 7
Nausea	72	48.3	6	4.0	70	46.7	4	2.7
Vomiting	63	42.3	7	4.7	63	42.0	6	4.0
Fatigue	27	18.1	2	1.3	11	7.3		0
Pyrexia	28	18.8	1	0.7	12	8.0		0
Anorexia	60	40.3	2	1.3	50	33.3	5	3.3
Alopecia	31	20.8	1	0.7	20	13.3		0

Data are number (%)

*AP* amrubicin/cisplatin, *EP* etoposide/cisplatin

Regarding cardiotoxicity, there was one case of ventricular arrhythmia and one of supraventricular tachyarrhythmia in the AP group and one case of myocardial ischemia in the EP group, all of which were reversible. The LVEF at baseline in the AP group was 65.8 ± 5.9 % (mean ± SD), while that post-treatment was 63.9 ± 5.2 %; hence, AP therapy had no clinically important effect. Severe AEs (SAEs) occurred in 21 patients in the AP group and eight in the EP group, but most were reversible. Although frequent SAEs in the AP group were grade 3–4 neutropenia and leukopenia, these were successfully treated with granulocyte colony-stimulating factor (G-CSF). Treatment-related death occurred in three patients (one with granulocytopenia, one with hypokalemia and cerebral infarction, and one with grade 4 myelosuppression) in the AP group and one (with acute cerebral infarction) in the EP group.

## Discussion

This is the first reported phase III study to compare AP therapy with EP therapy for previously untreated ED-SCLC. We demonstrated non-inferiority but not superiority of AP therapy to EP therapy, with a prolonged median OS of 1.5 months. It is conceivable that the effect of post-study treatment was minor, because the difference of median PFS between two groups was 1.1 months. In fact, approximately 75 % of the patients did not receive post-study treatment.

The toxicity of AP therapy was also tolerable, despite AE incidences in the AP group being higher than in the EP group. The most common severe toxicity associated with amrubicin was myelosuppression, but most cases were reversible. The rate of grade 3 or worse neutropenia was within the range of previous reports (95.1 % and 84.8 %) [22, 23], and the degree of myelosuppression and its risk of secondary serious infection and sepsis was manageable with protocol-specific dose reductions, treatment delays, and prophylactic use of G-CSF and antibiotics. The rate of febrile neutropenia in the AP group (4.0 %) was considerably lower than observed in a previous Japanese study by Satouchi et al. [29]. Although the reasons for this are not clear, almost 80 % of patients received G-CSF, and there were no differences between treatment groups in the use of G-CSF. This observation may be explained by the suitable use of G-CSF. No clinically significant LVEF reduction was found and there was no evidence of cardiomyopathy, congestive heart failure, or treatment-related cardiac mortality. While three patients in the AP group and one in the EP group died because of their treatment regimen, cancer chemotherapy is reported to be responsible for approximately 2–3 % of treatment-related deaths [30, 31]. Furthermore, there was no correlation between the number of administered treatment cycles and the frequency of treatment-related death risk in this study.

Recently, the West Japan Thoracic Oncology Group reported sequential chemotherapy consisting of three cycles of irinotecan and cisplatin followed by three cycles of amrubicin for previously untreated ED-SCLC [32]. This report was a phase II study but demonstrates the effective use of amrubicin in previously untreated SCLC.

Despite the high incidence of toxicity, amrubicin demonstrated sufficient efficacy compared with approved drugs for the treatment of SCLC. Its efficacy and alternate mechanism of action make it a potential candidate for treatment of this disease. More effective use of the evidence for amrubicin in the treatment of Chinese SCLC patients is needed.

## Conclusions

In our study, the OS of previously untreated Chinese patients with ED-SCLC following AP therapy was non-inferior to EP therapy, prolonging OS for 1.5 months. This result suggests that while AP therapy has sufficient efficacy, EP therapy is still the gold standard for first-line treatment of SCLC. Among the investigational drugs, amrubicin shows promise as a therapy for SCLC, and further studies are required to identify its most effective use.

### Ethics approval and consent to participate

All patients provided written informed consent. For a detailed list of the committees that granted ethical approval at each study site, please refer to the Supporting Information (Additional file 1.docx).

## Additional files

Additional file 1: List of ethics committees. Names of institutional ethics committees at each center. (DOCX 15 kb)

## Abbreviations

AE: Adverse events
AP: Amrubicin and cisplatin
CI: Confidence interval
ECOG PS: Eastern Cooperative Oncology Group performance status
ED: Extensive disease
ED-SCLC: Extensive-disease small-cell lung cancer
EP: Etoposide and cisplatin
FAS: Full analysis population
G-CSF: Granulocyte colony-stimulating factor
HR: Hazard ratio
LVEF: Left ventricular ejection fraction
MST: Median survival time
ORR: Overall response rate
OS: Overall survival
PFS: Progression-free survival
SAEs: Severe adverse events
SCLC: Small-cell lung cancer
