# Supplementary material for: Randomized phase III trial of amrubicin/cisplatin versus etoposide/cisplatin as first-line treatment for extensive small-cell lung cancer
Source: BMC Cancer. 2016 Apr 9;16:265. doi: 10.1186/s12885-016-2301-6 (PMC4826513; doi:10.1186/s12885-016-2301-6)
Supplement: Additional file 1: — List of ethics committees. Names of institutional ethics committees at each center. (DOCX 15 kb) [file 12885_2016_2301_MOESM1_ESM.docx]

**Supporting Information**

Additional file 2: List of ethics committees.

| Site Name | Ethics Committee |
| --- | --- |
| Cancer Hospital of Chinese Academy of Medical Sciences | Ethics Committee of Cancer Hospital of Chinese Academy of Medical Sciences |
| Jilin Cancer Hospital |  |
| Beijing Cancer Hospital |  |
| Xiangya Hospital, Central-South University |  |
| Beijing Chest Hospital |  |
| 307^th^ Hospital of the Chinese People’s Liberation Army |  |
| Peking Union Medical College Hospital of Chinese Academy of Medical Sciences |  |
| Jiangxi Provincial People’s Hospital |  |
| 81st Hospital of the Chinese People’s Liberation Army |  |
| West China Hospital, Sichuan University |  |
| Tangdu Hospital of the Fourth Military Medical University |  |
| Shanghai Changzheng Hospital |  |
| Jiangsu Cancer Hospital | Medical Ethics Committee of Jiangsu Cancer Hospital |
| Shanghai Chest Hospital | Ethics Committee of Shanghai Chest Hospital |
| Affiliated Cancer Hospital of Sun Yat-sen University | Ethics Committee of Affiliated Cancer Hospital of Sun Yat-sen University |
| The First Hospital of China Medical University | Medical Ethics Committee of The First Hospital of China Medical University |
| The First Hospital of Jilin University | Ethics Committee of The First Hospital of Jilin University |
